# ZIF-8 Nanoparticles Induce Behavior Abnormality and Brain Oxidative Stress in Adult Zebrafish (*Danio rerio*)

**DOI:** 10.3390/antiox12071345

**Published:** 2023-06-26

**Authors:** Liang Jin, Sijing Wang, Chen Chen, Xuchun Qiu, Chong-Chen Wang

**Affiliations:** 1 Key Laboratory of Estuarine Ecological Security and Environmental Health, Xiamen University Tan Kah Kee College, Zhangzhou 363105, China; jl002@xujc.com; 2 Institute of Environmental Health and Ecological Security, School of the Environment and Safety Engineering, Jiangsu University, Zhenjiang 212013, China; 3 Jiangsu Collaborative Innovation Center of Technology and Material of Water Treatment, Suzhou University of Science and Technology, Suzhou 215009, China; 4 Beijing Key Laboratory of Functional Materials for Building Structure and Environment Remediation, School of Environment and Energy Engineering, Beijing University of Civil Engineering and Architecture, Beijing 100044, China

**Keywords:** ZIF-8 nanoparticles, zebrafish, behavior, oxidative stress, toxicity

## Abstract

Zeolitic imidazolate framework-8 nanoparticles (ZIF-8 NPs) are typical metal–organic framework (MOF) materials and have been intensively studied for their potential application in drug delivery and environmental remediation. However, knowledge of their potential risks to health and the environment is still limited. Therefore, this study exposed female and male zebrafish to ZIF-8 NPs (0, 9.0, and 90 mg L^−1^) for four days. Subsequently, variations in their behavioral traits and brain oxidative stress levels were investigated. The behavioral assay showed that ZIF-8 NPs at 90 mg/L could significantly decrease the locomotor activity (i.e., hypoactivity) of both genders. After a ball falling stimulation, zebrafish exposed to ZIF-8 NPs (9.0 and 90 mg L^−1^) exhibited more freezing states (i.e., temporary cessations of movement), and males were more sensitive than females. Regardless of gender, ZIF-8 NPs exposure significantly reduced the SOD, CAT, and GST activities in the brain of zebrafish. Correlation analysis revealed that the brain oxidative stress induced by ZIF-8 NPs exposure might play an important role in their behavioral toxicity to zebrafish. These findings highlight the necessity for further assessment of the potential risks of MOF nanoparticles to aquatic species and the environment.

## 1. Introduction

Metal–organic frameworks (MOFs) are one of the most advanced nanomaterials assembled from metal ions or their clusters and organic linkers [1]. Due to their framework flexibility and huge surface areas, MOFs have been widely used in gas sorption, catalysis, fluorescence, proton conduction, and solar cells [2,3,4]. As a typical MOF material, zeolitic imidazolate framework-8 (ZIF-8) nanoparticles, constructed from Zn^2+^ ions and 2-methylimidazole ligands, have exceptional chemical and thermal stability. Thus, ZIF-8 nanoparticles (ZIF-8 NPs) have opened up many applications in oil–water separation, catalysis, and sensing techniques [5,6,7]. In recent years, the ZIF-8 NPs has emerged as a promising platform for drug delivery and controlled release [8]. Due to the increasing demand and production of those nanoparticles, an increasing risk of their release into the environment can be foreseen. Therefore, the potential risks of ZIF-8 NPs to organisms and the environment have attracted much attention.

It has been shown that ZIF-8 NPs exhibit in-vitro cytotoxicity. For example, ZIF-8 (0.028–5.6 mg L^−1^ as Zn) may increase ROS levels and cellular inflammation and eventually induce cytotoxicity to human hepatoma cells dose-dependently [9]. When human metastatic breast cancer cells, embryonic kidney cells, keratinocytes, mouse embryonic fibroblast cells, macrophage cells, and human osteoclast cells were exposed to ZIF-8 solution at >30 mg L^−1^, ZIF-8 was shown to potentially increase ROS production, which may activate apoptosis paths in all investigated cell lines [10]. Moreover, ZIF-8 nanoparticles at 12.5 and 25 mg L^−1^ can alter the actin organization and contractility of vascular smooth muscle cells [11]. Therefore, further exploration of the toxic effects of ZIF-8 is crucial for the safety evaluation of the large-scale application of ZIF-8.

In-vivo toxicity of nanoparticles has also been demonstrated in mammals and some aquatic organisms, and their toxicities are often closely related to oxidative stress. For example, intratracheal exposure to nickel oxide NPs (0.24 mg kg^−1^ for 6 weeks) may significantly alter the hydroxyl radicals, total superoxide dismutase (SOD), catalase (CAT), glutathione peroxidase (GPx), total antioxidant capacity, and lipid peroxidation in the liver of rats [12]. After intratracheal instillation, ytterbium oxide NPs can accumulate in the lung, heart, kidneys, and liver of mice, leading to oxidative stress, inflammation, and pathological damage [13]. Regarding aquatic species, our previous studies have shown that exposure to ZIF-8 NPs could induce oxidative stress in zebrafish larvae, accompanied by abnormal development and behavioral abnormalities [14]. Hu et al. [15] have reported that ZIF-8 nanoparticles induce neurobehavioral disorders through the regulation of ROS-mediated oxidative stress in zebrafish embryos, and Chen et al. [16] have found that oxidative damage was one of the main reasons for changing the behavioral responses to light-to-dark transitions in zebrafish larvae exposed to nanoplastics. Moreover, Yang et al. [17] also found that exposure to ZIF-8 NPs induced oxidative stress behaviors similar to the hormesis effect in the tissues of the Asian clam (*Corbicula fluminea* (O. F. Müller, 1774)). Thus, oxidative stress induced by nanoparticles can be considered one of the important mechanisms for their toxic effects.

Reactive oxygen species (ROS) are produced as the consequence of the normal aerobic physiological metabolism, and organisms also evolved antioxidant defense systems, e.g., SOD, CAT, GPx, glutathione-s-transferase (GST), and glutathione (GSH) to protect cells from attack by ROS [18]. When exposed to harmful stimuli, however, elevated intracellular ROS levels may exceed the ability of the antioxidant defense system to scavenge oxygen free radicals, resulting in oxidative stress [19]. It has been demonstrated that the oxidative stress effects of particulate pollutants exposure play an important role in their toxic effects on aquatic species [20,21]. Furthermore, oxidative stress induced by environmental pollutants is also of ecological significance [22]. For example, oxidative stress induced by pollutants could trigger behavioral impairments in the teleost, subsequently decreasing their fitness and survival [22,23]. However, little attention has been paid to the impacts of ZIF-8 NPs on adult fish behavior, let alone their association with oxidative stress.

Zebrafish (*Danio rerio*) is an ideal vertebrate model for ecotoxicology and drug safety assessment [24,25,26,27]. Many previous studies have used zebrafish behavioral changes to assess nanoparticle risks [28,29,30,31]. In this study, we exposed adult zebrafish to the suspension of ZIF-8 NPs and conducted behavior tests before and after a ball falling stimulation. Subsequently, responses in the brain levels of several oxidative stress parameters were examined. The objectives of this study were to investigate the impacts of ZIF-8 NPs on the behavioral traits and brain oxidative stress levels in adult zebrafish and to investigate the possible links between those responses.

## 2. Materials and Methods

### 2.1. Test Organism

Female and male zebrafish (AB-strain) were separately maintained in glass aquariums (containing 10 L of dechlorinated tap water) at 27 ± 1 °C and under a light:dark cycle of 14:10 h. The fish were fed newly hatched *Artemia* nauplii twice daily, and half of the water was changed every three days. The experiment followed the Guide for the Care and Use of Laboratory Animals (8th edition) by the National Research Council and obtained permission from the Jiangsu University Animal Care and Use Committee, Zhenjiang, China (Permit number SYXK (SU) 2018-0053).

### 2.2. Chemicals

The zeolitic imidazolate framework-8 nanoparticles (ZIF-8 NPs) were produced via the mechanochemical reaction [32] between ZnO and 2-methylimidazole (J&K Scientific LLC, San Jose, CA, USA) with a molar ratio of 1:2 and particle sizes of 100–400 nm [33]. Other chemical reagents (analytical grade) were purchased from Sinopharm Chemical Reagent Co., Ltd. (Shanghai, China). In addition, the enzyme-linked immunosorbent assay (ELISA) kits for assaying fish SOD, CAT, GST, and malonaldehyde (MDA) were purchased from Yanjin Biological Technology Co., Ltd. (Shanghai, China).

### 2.3. Experimental Design

Test solutions were prepared by pipetting calculated amounts of the ZIF-8 NPs stock suspension (100 mg mL^−1^ in distilled water) into dechlorinated tap water to obtain final concentrations of 0 (control), 9, and 90 mg L^−1^. Our previous study showed that the 96 h median lethal concentration (96 h-LC_50_) of ZIF-8 NPs for zebrafish larvae ranged from 483.3 to 915.2 mg L^−1^ [34]. In the present study, the exposure concentrations of ZIF-8 NPs were set to be approximately 1/100 and 1/10 of their highest 96 h LC_50_ value.

Females and males were exposed separately, with three replicates for each treatment (n = 3). For each replicate, five healthy fish were randomly selected and gently introduced into 3 L cylindrical glass tanks containing 2.0 L of a corresponding test solution. In this way, 45 females and 45 males were used for the exposure tests. The exposure was conducted under the conditions mentioned in Section 2.1, and continuous aeration was used for each beaker to prevent the aggregation and settling of ZIF-8 NPs. Following the guidelines for the testing of chemicals (OECD TG203) [35], the exposure period was set to be 4 days, and test solutions were renewed daily. The fish survival and state of test solutions were periodically observed, and the level of aeration was adjusted to ensure no obvious aggregates in solutions. After the 4-day exposure, behavioral traits were measured.

### 2.4. Behavioral Assay

For this assay, four zebrafish were selected and placed in an aquarium (20 cm × 9.5 cm × 10 cm) containing 1 L dechlorinated tap water. After a 10 min acclimation period, fish locomotion was tracked for 10 min using the DanioVision system (Noldus, The Netherlands). Subsequently, a startle behavior test was carried out following the method reported by Qiu et al. [36]. For this test, an iron ball was thrown 75 cm above the water (freefall to the center area of the aquarium), and then the ball was pulled up quickly by a string connected to it. Fish locomotion was tracked for 10 min immediately after the ball left the water surface. The behavioral traits were analyzed using EthoVision XT software (Vision 11.5; Noldus), and the locomotor activity was judged based on the threshold values reported by [37].

### 2.5. Measurement of Oxidative Stress-Related Biomarkers

Three hours after the behavioral test, the fish was placed into an ice water bath (0–4 °C, 10 min) for euthanasia [38]. Subsequently, the brain tissue was extracted and immediately frozen in liquid nitrogen and stored at −80 °C. For each replicate, 3 fish brains were pooled as one sample, and 3 replicates were used for each treatment group. The homogenate and supernatant were prepared following the method described by Qiu et al. [14]. Briefly, the weighted sample was homogenized with 9 vol (*w*/*v*) phosphate buffer saline (10 mM, pH 7.2–7.4) and centrifuged at 6000× *g* for 5 min. Subsequently, the supernatant was collected and used for the biological assay. The SOD, CAT, GST, and MDA levels in supernatants were detected using corresponding kits, which adopt the Sandwich-ELISA method. These ELISA kits use a capture antibody coated to the microplate and a horseradish peroxidase (HRP) conjugated antibody as the detection reagent, with TMB (3,3′,5,5′-tetramethyl benzidine) as the substrate. The optical density (OD) was measured using a microplate spectrophotometer (Synergy H4, BioTek, Winooski, VT, USA) at a wavelength of 450 nm. The concentrations of each antigen were calculated by comparing the OD of the samples to the standard curve. The protein concentrations in each supernatant were also measured using a BCA Kit (Bomei Biotechnology Co., Ltd., Hefei, Anhui Province, China), and the levels were normalized using the protein concentration (per mg-P).

### 2.6. Statistical Analysis

A generalized linear model (GzLM) was used to analyze the effects of ZIF-8 NPs, fish gender, and their interaction on the behavioral traits and the levels of oxidative stress-related biomarkers. Subsequently, a simple effects analysis was conducted to examine the difference between each exposure and control within either gender of fish. The correlations between the brain levels of oxidative stress-related biomarkers and behavioral parameters were analyzed by partial correlation analysis controlling for gender. All statistical analyses were performed using SPSS 16.0 (SPSS Inc., Chicago, IL, USA).

## 3. Results

There was no mortality in all the experimental groups during the 4-day exposure.

### 3.1. Impacts on the Normal Locomotor Behaviors

**Figure 1:**
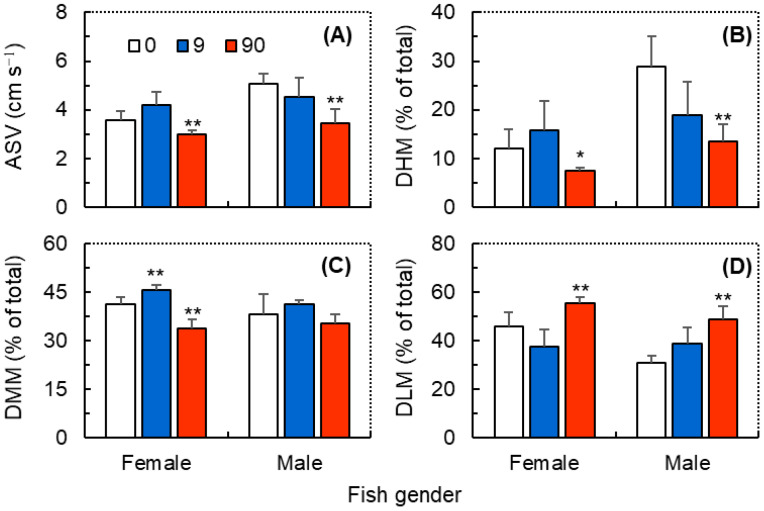
The locomotor behaviors of female and male zebrafish (*Danio rerio*) exposed to ZIF-8 NPs at 0 (control), 9.0, and 90 mg L^−1^. (**A**) average swimming velocity (ASV); (**B**) duration of high mobility (DHM); (**C**) duration of moderate mobility (DMM); (**D**) duration of low mobility (DLM). Data were measured within 10 min prior to the ball falling stimulation and are shown as mean ± SD (n = 3). Asterisks indicate significant differences between the exposure and control (* *p* < 0.05, ** *p* < 0.01).

As shown in Figure 1, exposure to ZIF-8 NPs tended to decrease the locomotor activity of both female and male zebrafish. The GzLM analysis showed that ZIF-8 NPs exhibited significant primary effects on the ASV, DHM, DMM, and DLM, and fish gender also exhibited significant primary effects on the ASV, DHM, and DLM of zebrafish. Moreover, their interaction effects on all of the abovementioned behavioral parameters were also significant (Table 1). Compared with the control, female zebrafish exposed to ZIF-8 NPs at 9.0 mg L^−1^ showed significantly higher DMM (Figure 1C), and those at 90 mg L^−1^ showed significantly lower ASV (Figure 1A), DHM (Figure 1B), and DMM (Figure 1C), with a significantly higher DLM value (Figure 1D). As for male zebrafish, exposure to ZIF-8 NPs at 9.0 mg L^−1^ did not induce significant variations in the behavioral traits related to the locomotor activity (Figure 1B), however, exposure at 9.0 mg L^−1^ induced significantly lower ASV (Figure 1A), DHM (Figure 1B), and FHM (Figure 1C), and also induced a significantly higher DHM (Figure 1D).

### 3.2. Impacts on Post-Stimulation Behaviors

**Figure 2:**
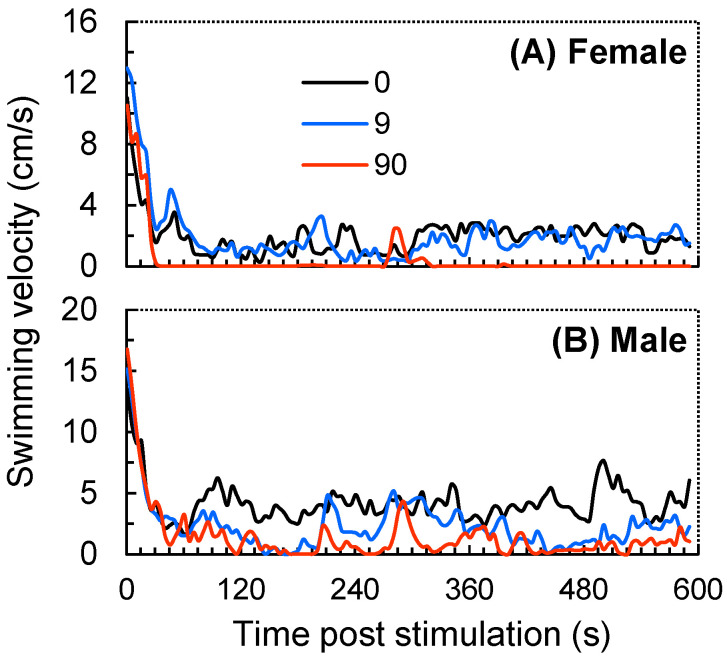
The time-dependent variation in the swimming velocity of female (**A**) and male (**B**) zebrafish exposed to ZIF-8 NPs at 0 (control), 9.0, and 90 mg L^−1^. Data were measured within 10 min post the ball falling stimulation and are shown as the average value of 4 fish within 5 s intervals.

**Figure 3:**
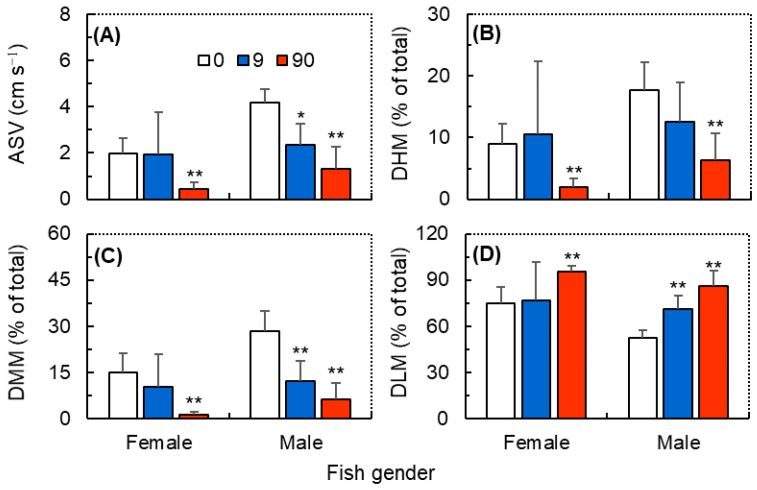
The post-stimulation behavioral traits of female and male zebrafish (*Danio rerio*) were exposed to ZIF-8 NPs at 0 (control), 9.0, and 90 mg L^−1^. (**A**) Average swimming velocity (ASV); (**B**) duration of high mobility (DHM); (**C**) duration of moderate mobility (DMM); (**D**) duration of low mobility (DLM). Data were measured within 10 min prior to the ball falling stimulation and are shown as mean ± SD (n = 3). Asterisks indicate significant differences between the exposure and control (* *p* < 0.05, ** *p* < 0.01).

After the ball falling stimulation, zebrafish exhibited temporary cessations of movement (i.e., freezing state) after a short period of high-speed swimming (Figure 2). The GzLM analysis showed that both ZIF-8 NPs and fish gender exhibited significant primary effects on all four post-stimulation behavioral traits, but their interaction effects were all insignificant (Table 1). Within the 10 min post stimulation, both female and male zebrafish exposed to ZIF-8 NPs at 90 mg L^−1^ exhibited significantly lower ASV (Figure 3A), DHM (Figure 3B), and DMM (Figure 3C), and a significantly higher DHM (Figure 3D), compared with the respective control group. However, exposure to ZIF-8 NPs at 9.0 mg L^−1^ only induced significantly lower ASV (Figure 3A) and DMM (Figure 3C), and a significantly higher DHM (Figure 3D) in male zebrafish but did not significantly affect those post-stimulation behavioral traits of females.

### 3.3. Impacts on Oxidative Stress-Related Bio-Markers

**Figure 4:**
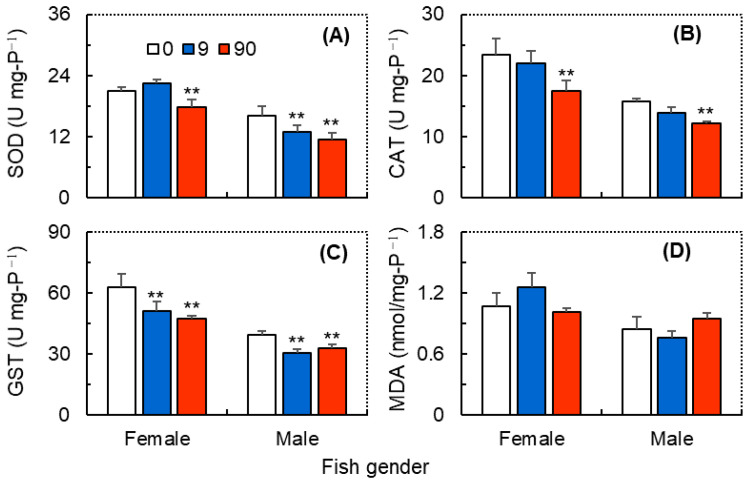
The brain levels of SOD (**A**), CAT (**B**), GST (**C**) and MDA (**D**) in female and male zebrafish (*Danio rerio*) exposed to ZIF-8 NPs at 0 (control), 9.0 and 90 mg L^−1^. Data are mean ± SD (n = 3), and asterisks indicate significant differences between the exposure and control (** *p* < 0.01).

As shown in Figure 4, ZIF-8 NPs could alter the activity of SOD, CAT, and GST in the brain of zebrafish, although no significant differences were observed in the brain MDA levels. The GzLM analysis showed that ZIF-8 NPs exhibited significant primary effects on the brain levels of SOD, CAT, and GST, and fish gender exhibited significant primary effects on the four parameters (Table 1). Moreover, their interaction effects on the brain levels of SOD, GST, and MDA were also significant (Table 1). As for female zebrafish, ZIF-8 NPs at 9.0 mg L^−1^ significantly decreased the GST activity, and exposure at 90 mg L^−1^ significantly decreased the activity of SOD, CAT, and GST (Figure 4A–C). As for male zebrafish, exposure to ZIF-8 NPs at 9.0 mg L^−1^ significantly decreased the activity of SOD (Figure 4A) and GST (Figure 4C), and at 90 mg L^−1^ significantly decreased the activity of SOD and GST (Figure 4A–C).

### 3.4. Correlation Analysis

The results of the partial correlation (controlled for gender) analysis are shown in Table 2. Regarding the correlations with the behavioral trait before stimulation, the SOD activity exhibited significant correlations with the ASV (positive), DHM (positive), and DLM (negative); and the CAT activity exhibited significant correlations with the ASV (positive), DHM (positive), DMM (positive) and DLM (negative) (Table 2). As to the correlations with the post-stimulation behavioral traits, both the SOD and CAT activities exhibited significant correlations with the ASV (positive), DHM (positive), DMM (positive) and DLM (negative), and the GST activity exhibited significant negative correlations with DMM (positive) and DLM (negative) (Table 2). Thus, the oxidative stress induced by ZIF-8 NPs seems to play an important role in their behavioral toxicity to zebrafish.

## 4. Discussion

Our results demonstrate that short-term exposure to ZIF-8 NPs could induce hypoactivity (90 mg L^−1^) in adult zebrafish and change their behavioral responses to stimulation (9 and 90 mg L^−1^). Behavior change is the intuitive expression of animals to environmental change, and environmental change can cause molecular changes that affect animal behavior [39,40,41,42]. As a typical model organism in toxicology and neurotoxicology, zebrafish have been widely used to study the neurobehavioral toxicity of nanoparticles. For example, Sarasamma et al. [21] have reported that exposure to C_70_ NP (0.5 and 1.5 mg L^−1^ for 2 weeks) could induce behavioral impairments in adult zebrafish, and Pitt et al. [43] have found that exposure to polystyrene NPs (1 mg L^−1^ for 6 days) could induce swimming hypoactivity in zebrafish larvae. However, Al-Ansari et al. [44] have reported that MIL-89 NPs (10–300 μM for 5 days) exhibit limited effects on the locomotion activity of zebrafish larvae. Thus, behavioral abnormalities seem to be typical responses of fish to nanoparticles, and are determined by the type and size of NPs, exposure concentration, duration, and fish growth stages [45,46,47,48]. Several recent studies have suggested that the behavioral toxicity of ZIF-8 NPs to zebrafish larvae is strongly affected by their exposure concentration and duration. For example, Hu et al. [15] have reported that ZIF-8 NPs (50–200 mg L^−1^ for 6 days) could significantly decrease the swimming speed of zebrafish larvae in a dose-dependent manner, Shi et al. [34] have reported that embryonic exposure to ZIF-8 NPs (9.0 mg L^−1^ for 5 days) significantly increased the locomotor activity of newly hatched zebrafish larvae, and Qiu et al. [33] have reported that embryonic exposure to ZIF-8 NPs (0.3 and 0.6 mg L^−1^ for 5 days) did not significantly alter the locomotor activity of zebrafish larvae. Furthermore, except for nontoxicity (i.e., nanosize effects), ZIF-8 NPs can also induce toxic effects by releasing Zn ions into exposure mediums [9,17,49]. The coexistence of particulate and ionic effects complicates its toxicity and thus emphasizes the need for more studies to assess their potential risk.

Spontaneous movement is essential for fish survival as it is a key factor for predation, migration, and predator avoidance [50,51,52]. Predation is one of the key factors governing patterns in natural systems, and adjustments of prey behaviors in response to a predator stimulus can have important ecological implications for wild animals [53,54,55]. Thus, we further designed a startle behavior test with a ball falling stimulation to examine the effect of ZIF-8 NPs on the behavioral responses of zebrafish. After the ball fell, zebrafish exhibited manic movement within the first 15 s and then showed a freezing state, resulting in relatively lower post-stimulation average swimming velocity. Compared with the control, zebrafish exposed to ZIF-8 NPs exhibited a longer duration of freezing state, suggesting that ZIF-8 NPs may alter their anti-predator strategy. In the face of threat (e.g., predators or other stimulation), fish may choose different strategies, including fleeing (quickly escaping), freezing (reducing activity) or maintaining a safe distance, depending on the type and degree of danger [53,54]. Although the freezing behavior may reduce the likelihood of being discovered by predators to some extent, choosing the most appropriate strategy is essential for fish to ensure their safety [54,55,56]. Nevertheless, it should be noted that the exposure concentrations of ZIF-8 NPs used in this study should be much higher than those in the environment. Further studies, such as the behavioral toxicity of ZIF-8 NPs at much lower concentrations (e.g., at μg L^−1^ and ng L^−1^ levels), are needed to assess their potential ecological risks in natural habitats.

Nanoparticles are known to induce oxidative stress by generating intracellular reactive oxygen species (ROS), which may provoke damage to multiple cellular organelles and processes [57]. Correspondingly, organisms also use antioxidant enzymes, such as SOD, CAT, and GST, to protect organisms against oxidative stress and lipid peroxidation [58,59]. In this study, exposure to ZIF-8 NPs significantly reduced the SOD, CAT, and GST activities in the brains of zebrafish. Previously, Saddick et al. [60] reported that exposure to zinc-NPs (2 mg L^−1^ for 15 days) significantly decreased the SOD, CAT and GST activity and gene expression in the brains of Nile tilapia (*Oreochromis niloticus* (Linnaeus, 1758)), and Afifi et al. [61] also found that silver-NPs (4 mg L^−1^ for 15 days) reduced the brain SOD, CAT and GST activity in redbelly tilapia (*Tilapia zillii* (Gervais, 1848)). Considering those antioxidant enzymes are important biochemical mediators of the antioxidant defense system, their decreased activities may further aggravate ROS damage. However, our results also suggest that ZIF-8 NPs exhibited limited effects on brain MDA levels. The MDA is one of the main products of lipid peroxidation and is considered a biomarker of oxidative damage in organisms [62]. A possible explanation for this phenomenon is that the ROS due to ZIF-8 NPs exposure may be scavenged by other antioxidant substances (e.g., thiol-containing enzymes and non-enzymatic antioxidants) or be used to produce other lipid peroxides (e.g., 4-hydroxynonenal and 4-hydroxyhexenal) [63]. Another possible explanation for the stable MDA content in zebrafish brains is the antioxidant properties of ZIF-8 NPs [64,65]. It has been reported that ZIF-8 NPs exhibit ROS scavenging capability in vitro and protect PC12 neuronal cells from apoptosis induced by free radicals, especially in the form of ZIF-8-capped CeO_2_ NPs [65]. Since Zn^2+^ released by nanoparticles is the main factor causing intracellular oxidative stress [9,17,49], ZIF-8 NPs may protect cells from oxidative damage due to their antioxidant ability, resulting in stable MDA levels.

Furthermore, our results confirm the linkage between decreased antioxidant enzyme levels and abnormal behavioral traits in zebrafish exposed to ZIF-8 NPs. Alteration in oxidative stress was the most common phenomenon in response to particulate pollutants, indicating it to be an important mechanism for inducing abnormal behavior in fish [20,66]. For example, Sarasamma et al. [21] have reported that exposure to C_70_ NP induced behavioral impairments in adult zebrafish, which could be correlated with changes in oxidative stress, inflammation, hypoxia, and imbalance of neurotransmitters in the brain. Recently, Hu et al. [15] have reported that ROS-mediated oxidative stress is putatively involved in neurotoxicity and behavioral dysfunction in zebrafish exposed to ZIF-8 NPs. However, Hu et al. [15] have also observed that ZIF-8 NPs (50–200 mg L^−1^ for 6 days) significantly increase the ROS, SOD, CAT, and MDA levels in zebrafish larvae, contrary to our findings. We infer that ZIF-8 NPs might exhibit dual effects on the oxidative stress state in zebrafish, which is determined by the exposure concentration, duration, and growth stages. Therefore, further studies are necessary to establish the exact mechanisms underlying the oxidative stress and behavioral toxicity induced by ZIF-8 NPs.

## 5. Conclusions

Our findings suggest that exposure to ZIF-8 NPs could induce hypoactivity in adult zebrafish and change their behavioral responses to stimulation. After a ball falling stimulation, males exposed to ZIF-8 NPs at 9.0 and 90 mg L^−1^ exhibited more freezing states than those in control, resulting in significantly lower locomotor activity. In contrast to males, the post-stimulation locomotor activity of females exposed to ZIF-8 NPs at 9.0 mg L^−1^ did not alter significantly. Thus, female zebrafish seem less sensitive to ZIF-8 NPs than males, although the post-stimulation locomotor activity of females was also significantly decreased at a ten times higher concentration (i.e., 90 mg L^−1^). Regardless of gender, ZIF-8 NPs significantly reduced the SOD, CAT, and GST activities in the brain of zebrafish. Correlation analysis revealed that variations in the activities of these antioxidant enzymes might play important roles in the behavioral impacts of ZIF-8 NPs on adult zebrafish. Therefore, further studies on the potential risks of MOF nanoparticles are still needed to make it a safer material for the environment and for humans.

## Figures and Tables

**Table 1:** Summary of generalized linear model testing the statistical significance for the effect of zeolitic imidazolate framework nanoparticles (ZIF-8 NPs), fish gender (FG), and their interaction (ZIF-8 NPs × FG) on the behavioral traits and brain levels of oxidative stress-related biomarkers in zebrafish (*Danio rerio*) ^1^.

	Normal Behaviors				Post-Stimulation Behaviors				Oxidative Stress Biomarkers			
	ASV	DHM	DMM	DLM	ASV	DHM	DMM	DLM	SOD	CAT	GST	MDA
ZIF-8 NPs (*df* = 2)	37.7 **	32.3 **	77.3 **	56.7 **	49.0 **	31.2 **	52.4 **	54.1 **	45.6 **	38.3 **	59.0 **	0.87
FG (*df* = 1)	14.7 **	20.1 **	2.45	11.1 **	9.45 *	4.50 *	6.96 **	6.75 **	181.2 **	125.3 **	216.2 **	45.8 **
Interaction (*df* = 2)	9.13 *	9.2 *	7.89 *	9.53 **	5.84	2.14	3.65	3.84	14.9 **	4.25	7.89 **	27.4 **

^1^ Type III Wald chi square is listed, and asterisks indicate significance (* *p* < 0.05; ** *p* < 0.01). Abbreviations: *df*: degree of freedom; SOD: superoxide dismutase; GST: glutathione S-transferase; CAT: catalase; MDA: malondialdehyde; ASV: average swimming velocity; DHM: duration of high mobility; DMM: duration of moderate mobility; DLM: duration of low mobility.

**Table 2:** Partial correlation (controlled for gender) between the brain level of oxidative stress-related parameters and the behavioral traits of zebrafish (*Danio rerio*) ^1^.

	Behavioral Trait before Stimulation				Post-Stimulation Behavioral Traits			
	ASV	DHM	DMM	DLM	ASV	DHM	DMM	DLM
SOD	0.770 **	0.808 **	0.420	−0.808 **	0.680 **	0.657 **	0.624 **	−0.663 **
CAT	0.557 *	0.587 *	0.486 *	−0.660 **	0.712 **	0.650 **	0.746 **	−0.748 **
GST	0.261	0.380	0.075	−0.287	0.565 *	0.474	0.652 **	−0.579 **
MDA	0.051	0.030	0.253	−0.144	−0.133	−0.200	−0.052	0.142

^1^ Partial correlation coefficients are listed, and asterisks indicate a significant correlation (* *p* < 0.05; ** *p* < 0.01). Abbreviations: SOD: superoxide dismutase; CAT: catalase; GST: glutathione *S*-transferase; MDA: malondialdehyde; ASV: average swimming velocity; DHM: duration of high mobility; DMM: duration of moderate mobility; DLM: duration of low mobility.

## Data Availability

Data is contained within the article.
